# Experience of violence and self-rated health: Do youths disclose their experiences when visiting a Youth Centre in Sweden

**DOI:** 10.1177/1403494820921690

**Published:** 2020-05-26

**Authors:** Carina Petersson, Katarina Swahnberg, Ulla Peterson, Marie Oscarsson

**Affiliations:** Department of Health and Caring Sciences, Linnaeus University, Kalmar, Sweden

**Keywords:** Self-rated health, Sweden, violence, youth, Youth Centre

## Abstract

*Background*: Being exposed to violence is a global health problem, increasing the risk of suffering from ill health. The main aim of this study was to estimate the prevalence of emotional, physical and sexual violence victimisation and its association to self-rated health among youths. The second aim was to investigate whether the youths had disclosed to healthcare professionals at a Youth Centre or others about being exposed. *Methods*: The cross-sectional study includes data from a web survey of youths, aged 15–25 (*n*=500), collected in Sweden. Descriptive statistics and univariate analyses were used for the analyses. *Results*: In all, emotional, physical or sexual violence during their lifetime was reported by 43.2% and 22.8% of youths during the last year. In total, 88% of the respondents assessed their self-rated health as good, very good or excellent. Those who had been exposed to emotional, physical or sexual violence during their lifetime reported statistically significant lower self-rated health (fair and poor) than those who were not victimised. When healthcare professionals at the Youth Centre asked youths about exposure during their lifetime, one-fifth disclosed having been exposed. ***Conclusions*: Youths who reported any type of violence during their lifetime showed lower self-rated health compared to those who were not exposed. Youth Centres have an important role in identifying youths who are exposed to violence and/or self-report their health as low. Still, only a minority of youths who have been exposed to violence told health professionals at a Youth Centre about it when asked. It is necessary to further investigate how the issues can be best addressed.**

## Background

The World Health Organization (WHO) states that interpersonal violence is a global public health problem [1, 2], and the risk of exposure in Sweden is highest among youths [3]. In Sweden, the National Board of Health and Welfare (NBHW) describe interpersonal violence as a combination of emotional, physical and sexual violence (EPS). Multi-country studies state that the prevalence of violence among women aged 15–24 years old varies between 1.2% and 66% [4, 5]. Palm reported that 56% of youths stated they had experienced EPS or witnessed violence in a Swedish study [6].

There are gender differences in what type of violence is reported as most common by women and men [7, 8] and being exposed to violence affects youth health [1]. The WHO stated that violence causes ill health and human suffering, disabilities and deaths [1]. International studies show a strong association between women’s ill health and experience of violence [4, 9]. Swedish studies show that those exposed suffer from more anxiety, depression, poor mental health and self-harm compared to those who are not victimised [6, 10, 11].

To follow the public health status, a one-item question, self-rated health (SRH) is frequently used. SRH is useful for epidemiological investigations [12] and has been found to be associated with morbidity and mortality [13, 14]. Early in life, SRH is also useful as a predictor for staying healthy and contributing to a more salutogenic rather than pathogenic perspective [15, 16]. Individuals’ states of SRH may influence SRH across many years [17, 18] and in general, women stated lower SRH than men [19, 20]. DeSalvo et al. [14] described an association between poor SRH and higher mortality risk in relation to those who reported excellent SRH.

Few studies have examined the relationship between exposure to violence and SRH. Boynton-Jarrett et al. [21] showed that cumulative exposure was associated with risk of poor SRH, and Blom et al. [20] showed that experience of multiple-violence victimisation was associated with lower SRH.

Although enquiring during the healthcare visit as to whether the individual has experienced violence is deemed to be a sensitive topic, it is necessary to do so [22]. This will allow an opportunity to offer help to those in need. In Sweden, it is mandatory for healthcare professionals to report it to social services if they meet anyone <18 years old who has been exposed to violence. The NBHW also recommends that healthcare professionals routinely ask youths about being exposed as they often seek healthcare for other symptoms. Consequently, this has been implemented at most Youth Centres (YC) in Sweden. The staff at a YC include a midwife, a counsellor/psychologist and a physician to achieve comprehensive care. The YCs’ goal is to promote physical and mental health, with a focus on sexual and reproductive health and rights among youths.

However, there can be obstacles that prevent youths from reporting violence or healthcare professionals from asking questions. Leander et al. [3] and Lemaigre et al. [23] reported that most victims want to keep information about having been victimised a secret as it is often associated with guilt and shame. Lack of knowledge, guidelines and/or routines or lack of time during visits [24] can determine whether healthcare professionals ask youths about having been victimised.

Previous studies have shown large variations between youths’ exposure and type of violence reported by women and men. Exposure to EPS affects youth health, but there is limited research about the association between the combination of EPS and SRH. In addition, there is limited research on whether youths who have been exposed to violence have disclosed this to healthcare professionals at YCs.

## Aims

The first aim of this study was to estimate the prevalence of emotional, physical and sexual violence victimisation and its association to self-rated health among youths who visit a YC in Sweden. The second aim was to investigate whether the youths had disclosed to healthcare professionals at a YC or others about being exposed to violence.

## Methods

This cross-sectional study is based on web surveys distributed to youths during their visit at a YC in south-eastern Sweden and is part of a larger project. Other articles in the project highlight the relationship between sexual health and violence as well as the description of youths’ own experiences of violence. The study was approved by the Regional Ethical Review Board of Linköping (Dnr 2015/ 245-31).

### Sample and procedure

Youths, women (15–23 years) and men (15–25 years) visiting one YC in Sweden were consecutively recruited. Exclusion criteria were mental retardation and/or lack of proficiency in the Swedish language. Data collection was performed over 1 year, from November 2015 to November 2016. In total, 4457 youths visited the YC during this time and were eligible for participation: 3919 (87.9%) women and 538 (12.1%) men. The healthcare professionals informed youths about the study at the end of their visit to the YC and no incentives were given for answering the web survey. Those who were willing to participate received written information before answering the web survey in a quiet room. The survey was online and self-completed, available in the Swedish language. Data were stored on a server and transferred to IBM SPSS Statistics version 24.0.

### Web survey

This study presents the responses from 25 of the total 52 web survey questions. The remainder of the questions are presented in other studies. The web survey was constructed by the research team based on experience and knowledge from previous studies [25], and well-established instruments: NorVold Abuse Questionnaire (NorAQ) [26, 27] and SRH [28, 29]. Socio-demographic factors were assessed using nine questions. In addition, youths were asked two questions: first, whether they had reported their exposure to staff at YC and, second, if they had reported it to others, such as police, staff at school, other healthcare professionals or friends. Finally, a question was asked about the youths’ perceptions of responding to questions about violence.

**Figure 1.**
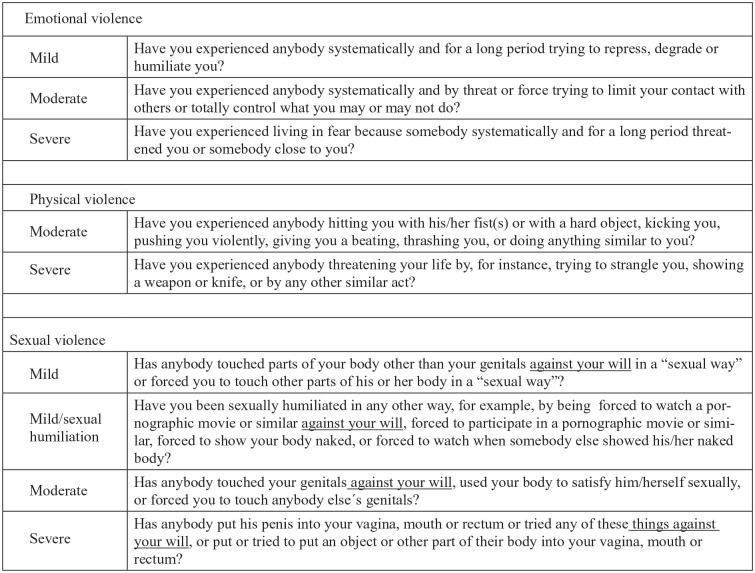
Questions about exposure to interpersonal violence in NorVold Abuse Questionnaire (NorAQ).

Exposure to violence was measured using the NorAQ [26, 27]; hence, all questions included about violence are validated. Experiences of each type of EPS were defined by nine questions based on one or more positive answers to the questions that specify each type of violence (Figure 1). The questions included severity of violence behaviour, ranging from mild, moderate to severe. Each type or severity of violence had the same response alternatives: no, yes during childhood <18 years, yes during adulthood ⩾ 18 years, or yes to both childhood and adulthood. NorAQ allows for an approximate classification according to the degree of severity of the abusive act. One question for each type of violence measured if the respondent had been exposed to violence during the last year, with no/yes as the possible answer. The question of mild physical violence was excluded in our study because a previous study [27] showed considerably lower concurrent validity than the other questions.

The likelihood ratio among women/men was 38/3 for emotional, 6/9 for physical and 42/46 for sexual violence. The lower estimate for physical violence was mainly ascribed mild physical violence capturing also minor abusive events.

SRH was measured by one item from the Short Form Health Survey (SF-36) [28, 29]: ‘In general, how would you describe your health?’ The item has a standardised response format with five alternatives ranging from ‘1=excellent, 2=very good, 3=good, 4=fair to 5=poor’. The response alternatives fair and poor were considered as low SRH. The focus was the individual’s own estimation of health.

### Data analysis

Relevant descriptive statistics and univariate analyses were used for the analyses.

The two degrees of mild (no contact) and mild2 (no genital contact) sexual violence were merged and referred to as mild. If a respondent reported several degrees of a specific type of violence, they were classified according to the most severe act of violence. NorAQ covers EPS lifetime and the last 12 months (year). To describe co-occurrence between EPS victimisation, a new variable was created. When summing the value, zero stands for no violence and values one to three indicate one, two or three types of violence during lifetime. A Mann-Whitney *U*-test was used to investigate the difference between sexes and prevalence of EPS, difference in SRH between those who had been exposed and those who had not, as well as differences between sex and those who had disclosed about exposure. Data were analysed using IBM SPSS Statistics version 24.0.

## Results

A total of 507 youths, aged 15–25, answered the web survey. Seven youths were excluded as they did not match the inclusion criteria and in all 500 youths participated. Of those, 89.4% (*n*=447) were women and 10.6% (*n*=53) were men. A majority, 96% (*n*=480), were born in Sweden and 90% (*n*=449) assessed their household economy as good or very good. Socio-demographic factors are presented in Table I. Most of the youths 89% (*n*=443) stated they had a scheduled appointment at a YC before they answered the web survey.

**Table I.** Sociodemographic factors by sex (*N*=500).

% (*n*)	Female	Male	Total
**Sex**	89.4 (447)	10.6 (53)	100 (500)
**Age**			
< 18 years	21.9 (98)	7.5 (4)	20.4 (102)
⩾ 18 years	78.1 (349)	92.5 (49)	79.6 (398)
**Occupation**			
Studying	69.1 (309)	52.8 (28)	67.4 (340)
Working	22.8 (102)	41.5 (22)	24.8 (124)
Unemployed	6.1 (27)	3.8 (2)	5.8 (29)
SA/parental leave/other	1.3 (6)	1.9 (1)	1.4 (7)
**Living arrangements**			
Alone	20.6 (92)	35.8 (19)	22 (111)
With parents/foster parents	55.7 (249)	49.1 (26)	55 (275)
At a family home/institution	0.4 (2)	0	0.4 (2)
With friend/friends	4.5 (20)	3.8 (2)	4.4 (22)
With partner	15.7 (70)	7.5 (4)	14.8 (74)
Other	2.2 (10)	3.8 (2)	2.4 (12)
**Highest level of education**			
Not complete/incomplete primary school	3.6 (16)	0	3.2 (16)
Elementary school	26.6 (119)	15.1 (8)	25.4 (127)
High school	62.4 (279)	67.9 (36)	63 (315)
Folk high school/vocational training	2.5 (11)	3.8 (2)	2.6 (13)
University/college graduate	4.3 (19)	13.2 (7)	5.2 (26)

SA: sickness absence.

Internal dropout = 0.2–0.8%.

Prevalence of EPS is presented in Table II. In total, 43.2% (*n*=216) of the youths reported any type of violence victimisation during lifetime, and 22.8% (*n*=114) answered they were exposed to violence during the last year. Among men, 47.2% (*n*=25) reported they had been exposed to violence during lifetime, and corresponding frequency for women was 42.7% (*n*=191). For men, experience of physical violence was the most common over their lifetime, 35.9% (*n*=19), and the last year, 13.2% (*n*=7). Emotional violence was the most common for women, in their lifetime 27.6% (*n*=123) and in the last year, 11.9% (*n*=53). There was a statistically significant difference between the sexes regarding exposure to physical violence (*p*< 0.001).

**Table II.** Prevalence of emotional, physical and sexual violence (*N*=500).

(% (*n*))	Emotional violence			Physical violence			Sexual violence		
	Women	Men	Total	Women	Men	Total	Women	Men	Total
	*n*=447	*n*=53	*N*=500	*n*=447	*n*=53	*N*=500	*n*=447	*n*=53	*N*=500
No	72.5 (324)	69.8 (37)	72.2 (361)	86.1 (385)	64.2 (34)	83.8 (419)	74.3 (332)	96.2 (51)	76.6 (383)
Severity									
Mild									
<18 years	7.2 (32)	7.5 (4)	7.2 (36)				2.4 (11)	1.9 (1)	2.4 (12)
⩾18 years	1.3 (6)	1.9 (1)	1.4 (7)				5.1 (23)	0	4.6 (23)
Both	0.9 (4)	5.7 (3)	1.4 (7)				1.1 (5)	0	1.0 (5)
Lifetime	9.4 (42)	15.1 (8)	10.0 (50)				8.6 (39)	1.9 (1)	8.0 (40)
Moderate									
<18 years	4.3 (19)	7.5 (4)	4.6 (23)	5.8 (26)	9.4 (5)	6.2 (31)	2.9 (13)	0	2.6 (13)
⩾18 years	3.4 (15)	0	3.0 (15)	1.1 (5)	13.2 (7)	2.4 (12)	2.2 (10)	0	2.0 (10)
Both	0.9 (4)	0	0.8 (4)	0.7 (3)	0	0.6 (3)	0.4 (2)	0	0.4 (2)
Lifetime	8.6 (38)	7.5 (4)	8.4 (42)	7.6 (34)	22.6 (12)	9.2 (46)	5.5 (25)	0	5.0 (25)
Severe									
<18 years	5.8 (26)	5.7 (3)	5.8 (29)	4.3 (19)	5.7 (3)	4.4 (22)	6.0 (27)	0	5.4 (27)
⩾18 years	3.4 (15)	0	3.0 (15)	1.8 (8)	3.8 (2)	2.0 (10)	4.5 (20)	0	4.2 (21)
Both	0.4 (2)	1.9 (1)	0.6 (3)	0.2 (1)	3.8 (2)	0.6 (3)	0.9 (4)	1.9 (1)	0.8 (4)
Lifetime	9.6 (43)	7.5 (4)	9.4 (47)	6.3 (28)	13.3 (7)	7.0 (35)	11.4 (51)	1.9 (1)	10.4 (52)
Any violence past year	11.9 (53)	7.5 (4)	11.4 (57)	7.6 (34)	13.2 (7)	8.2 (41)	11 (49)	3.8 (2)	10.2 (51)
Any lifetime violence	27.6 (123)	30.1 (16)	27.8 (139)	13.9 (62)	35.9 (19)	16.2 (81)	25.5 (115)	3.8 (2)	23.4 (117)

Internal dropout: 2–3.4%.

For the group of youths <18 years (*n*=102), 22.5% (*n*=23) said they had been exposed to violence during last year and 35.3% (*n*=36) during their lifetime.

Of those youths (*n*=216) who reported exposure during their lifetime, 55.1% (*n*=119) stated one type of violence, 33.8% (n=73) two types and 11.1% (*n*=24) replied they had been exposed to all three types of violence. Moreover, 28.3% (*n*=15) of men and 23.3% (*n*=104) of women expressed being exposed to one type of violence. Also, 15.1% (*n*=8) of men and 14.5% (*n*=65) of women reported two types of violence. Exposure to all three types of violence was stated by 4.9% (*n*=22) of women and 3.8 (*n*=2) of men.

In all, 88% (*n*=441) of youths assessed their SRH as good, very good or excellent. A larger proportion of women (11.9%) reported lower SRH (fair or poor) compared to men (5.7%). Those youths who had been exposed to any type of violence during their lifetime (*n*=216) showed a statistically significant lower SRH (*p*= 0.002) than those who were not victimised. Lower SRH was reported by those who had been exposed to emotional violence (*p*= 0.002), physical violence (*p*= 0.03) and sexual violence (*p*= 0.01) during their lifetime, compared with those who had not. There was no statistically significance difference in SRH between those who reported exposure to any type of violence and non-violence, during the last year (Table III).

**Table III.** Prevalence among youths reporting SRH with EPS, EPS during last year, EPS during lifetime and non-violence (*N*=500).

% *(n)*	Low SRH	High SRH
Emotional violence *n*=139	16.5 (23)	83.5 (116)
Non-emotional violence *n*=361	9.2 (33)	90.8 (325)
Physical violence *n*=81	19.8 (16)	80.2 (65)
Non-physical violence *n*=419	9.6 (40)	90.4 (376)
Sexual violence *n*=117	17.9 (21)	82.1 (96)
Non-sexual violence *n*=383	9.2 (35)	90.8 (345)
EPS last year *n*=114	12.2 (14)	86.8 (99)
Non-EPS year *n*=384	10.9 (42)	89.1 (342)
EPS lifetime *n*=216	16.2 (35)	83.8 (181)
Non-EPS lifetime *n*=281	7.5 (21)	92.5 (260)

EPS: emotional, physical and/or sexual violence; SRH: self-rated health.

Internal dropout: 0.6%.

Response alternatives fair and poor were considered as low SRH.

Of those youths who reported any type of violence during their lifetime (*n*=216), in total 38% (*n*=82) had disclosed it to health professionals at a YC and/or others, such as police, staff at school, and/or other professionals in healthcare or friends. Disclosure at YC or to others about having been exposed to violence during the last year and their lifetime is presented in Table IV.

**Table IV.** Prevalence among youths who told the health professionals at a Youth Centre or others about emotional, physical and/or sexual violence during last year and/or lifetime (*N*=216).

% (n)	Told health professionals at Youth Centre about violence	Told police/ staff at school/ other health professionals in healthcare/ friends about violence
Emotional violence		
Last year (*n*=57)	26.3 (15)	42.1 (24)
Lifetime (*n*=139)	22.3 (31)	36.7 (51)
Physical violence		
Last year (*n*=41)	24.4 (10)	34.1 (14)
Lifetime (*n*=81)	25.9 (21)	40.7 (33)
Sexual violence		
Last year (*n*=51)	27.5 (14)	45.1 (23)
Lifetime (*n*=117)	25.6 (30)	45.3 (53)
Total (*N*=216)	21.8 (47)	35.6 (77)

Internal dropout: 9.3–9.7%.

In all, youths who had been exposed to sexual violence during the last 12 months 27.5% (*n*=14) reported the highest number of disclosures at YC. Sexual violence during their lifetime was the type of violence that most youths, 45.3% (*n*=53), disclosed to police or staff at school, other health professionals and/or friends. There was no statistically significant difference between the sexes regarding having disclosed exposure to violence to health professionals at YC. More women (16.3%) than men (7.5%) had told police/staff at school/other health professionals (not in the YC) or friends about violence (*p*= 0.025).

Of those youths who reported EPS during their lifetime and stated low SRH (*n*=35), 40% (*n*=14) disclosed both at a YC and to others of having been exposed to violence. Regarding exposure to violence during the last year and those youths who stated low SRH (*n*=14), 50% (*n*=7) had disclosed it (both at YC and to others).

Overall, 91% (*n*=453) of youths did not perceive the questions about violence in this study as unpleasant to answer, 85% (*n*=424) did not find the questions difficult to answer and 61% (*n*=306) felt that the web survey was important.

## Discussion

The findings in this study showed a high prevalence of EPS among youths who visited a YC. Only a fifth of those who stated having been exposed to EPS disclosed it to health professionals at a YC when asked. Those who were exposed during their lifetime reported statistically significant lower SRH than those who were not victimised.

### Exposure of EPS

Measuring prevalence of EPS is considered complex and it may be difficult to compare results in different studies depending on which definition, sampling strategies and data collection procedures are used. The prevalence of violence in our results is, however, consistent with previous studies in Sweden [3, 8]. Also, the gender differences we found in the type of violence that women and men were exposed to is in line with other studies [7, 8].

### Disclosure about EPS

By screening for violence in healthcare, exposed individuals can be identified at an early stage and offered support and help if required. In this study, 21.8% of those youths who had been exposed to violence disclosed it to health professionals at a YC, where there is routine screening for violence. This is a low figure, and previous studies propose possible reasons such as guilt and shame among victims or lack of guidelines and/or time during visit [3, 23, 24]. In our study, 89% of the youths had scheduled an appointment; thus, time should be allocated to address the issue. However, it is also of importance to ask those who visit the YC without a scheduled appointment about exposure to violence. Another possible reason for not disclosing their exposure to violence might be that it happened several years ago and therefore the individuals do not feel that it is important to talk about it again. Some youths may already have received needed assistance elsewhere, thus not needed to disclose the violence when visiting the YC. This is confirmed in our results, as 35.6% indicated they had previously told others about the exposure to violence. Nonetheless, even though youths may have told others beforehand, it is important the question is asked at a YC to determine whether exposure to violence continues to affect them. If youths disclose having been victimised when asked, healthcare professionals at YCs have the opportunity to offer personalised assistance to those in need.

### Measuring SRH

In all, low SRH was stated by 12% of youths in the present study, and those youths who reported exposure during their lifetime stated a statistically significant lower SRH compared with those who were not victimised. It is important to pay attention to these results as previous studies indicate SRH that is stable for a long time can be used as a predictor among youths, both in ability to stay healthy or morbidity and mortality [13–18]. To provide an opportunity to identify youths with low SRH, the question of SRH should be asked during visits to a YC. This will allow healthcare professionals provide support for health promotion and/or prevent future ill health in youths. In our study, a higher degree of women reported low SRH compared to men, which corresponds with other studies [19, 20].

Future studies should focus on *youths’ own experience* of being exposed to violence and whether this has affected their life or health, what kind of support they may need, as well as how the question about violence should be presented most appropriately.

## Methodological considerations

The main limitations of this study were that the survey was only available in Swedish language and there was a small sample in relation to the total number of visitors. We were told that no interpreter was registered among the visitors during data collection but we do not know about the number of visitors that did not understand or read Swedish. The reasons for the small sample could be twofold: first, some youths had taken leave from school and did not want to spend more time doing the web survey. Second, during the time for data collection, the YC had shortage of midwives who were occasionally replaced by temporary staff who were not as familiar with the web survey as the regular staff. Another limitation is that we do not know how often the violence occurred or who the perpetrators were. One strength is that the proportion of men versus women is similar for all visitors at the YC over 1 year. Another strength is the use of validated instruments and the low internal dropout rate, which increases the reliability and validity of the results.

The result showed that youths exposed to EPS during their lifetime rated their health as worse compared to the non-victimised youths, which underlines the importance of asking about SRH as a complement to the question about exposure to violence. The low number of youths who stated exposure to any type of violence during the last year and low SRH limits the statistical power. However, SRH might be influenced by several different factors such as psychological, medical and social circumstances [30] that have not been controlled for in the present study. This is a limitation and the results should therefore be interpreted with caution and need to be further examined in studies adjusting for other possible factors that might affect SRH.

## Conclusions

Exposure to EPS among youths visiting YC is high. Enquiring about whether one has been exposed to violence as a matter of routine is apparently important as those youths who reported having been exposed judged their SRH as statistically significantly lower than those who were not victimised. By identifying both SRH and exposure to violence among youths, there is an opportunity, at an early stage, to offer support or help to those in need.
